# Supplementary material for: First close insight into global daily gapless 1 km PM2.5 pollution, variability, and health impact
Source: Nat Commun. 2023 Dec 15;14:8349. doi: 10.1038/s41467-023-43862-3 (PMC10724144; doi:10.1038/s41467-023-43862-3)
Supplement: Supplementary file 1 — Supplementary Information [file 41467_2023_43862_MOESM1_ESM.pdf]

Supplementary Information for

First close insight into global daily gapless 1 km $PM_{2.5}$ pollution, variability, and health impact

Jing Wei[1*], Zhanqing Li[1*], Alexei Lyapustin[2], Jun Wang[3], Oleg Dubovik[4], Joel Schwartz[5], Lin Sun[6], Chi Li[7], Song Liu[8], and Tong Zhu[9]

1. Department of Atmospheric and Oceanic Science, Earth System Science Interdisciplinary Center, University of Maryland, College Park, MD, USA
2. Laboratory for Atmospheres, NASA Goddard Space Flight Center, Greenbelt, MD, USA
3. Department of Chemical and Biochemical Engineering, Iowa Technology Institute, The University of Iowa, Iowa City, IA, USA
4. Laboratoire d'Optique Atmosphérique, Université de Lille, CNRS, Lille, France
5. Department of Environmental Health, Harvard TH Chan School of Public Health, Boston, MA, USA
6. College of Geodesy and Geomatics, Shandong University of Science and Technology, Qingdao, China
7. Department of Energy, Environmental and Chemical Engineering, Washington University in St. Louis, St. Louis, Missouri, USA
8. School of Environmental Science and Engineering, Southern University of Science and Technology, Shenzhen, China
9. State Key Joint Laboratory of Environmental Simulation and Pollution Control, College of Environmental Sciences and Engineering, Peking University, Beijing, China

*Corresponding authors:
zhanqing@umd.edu; weijing_rs@163.com

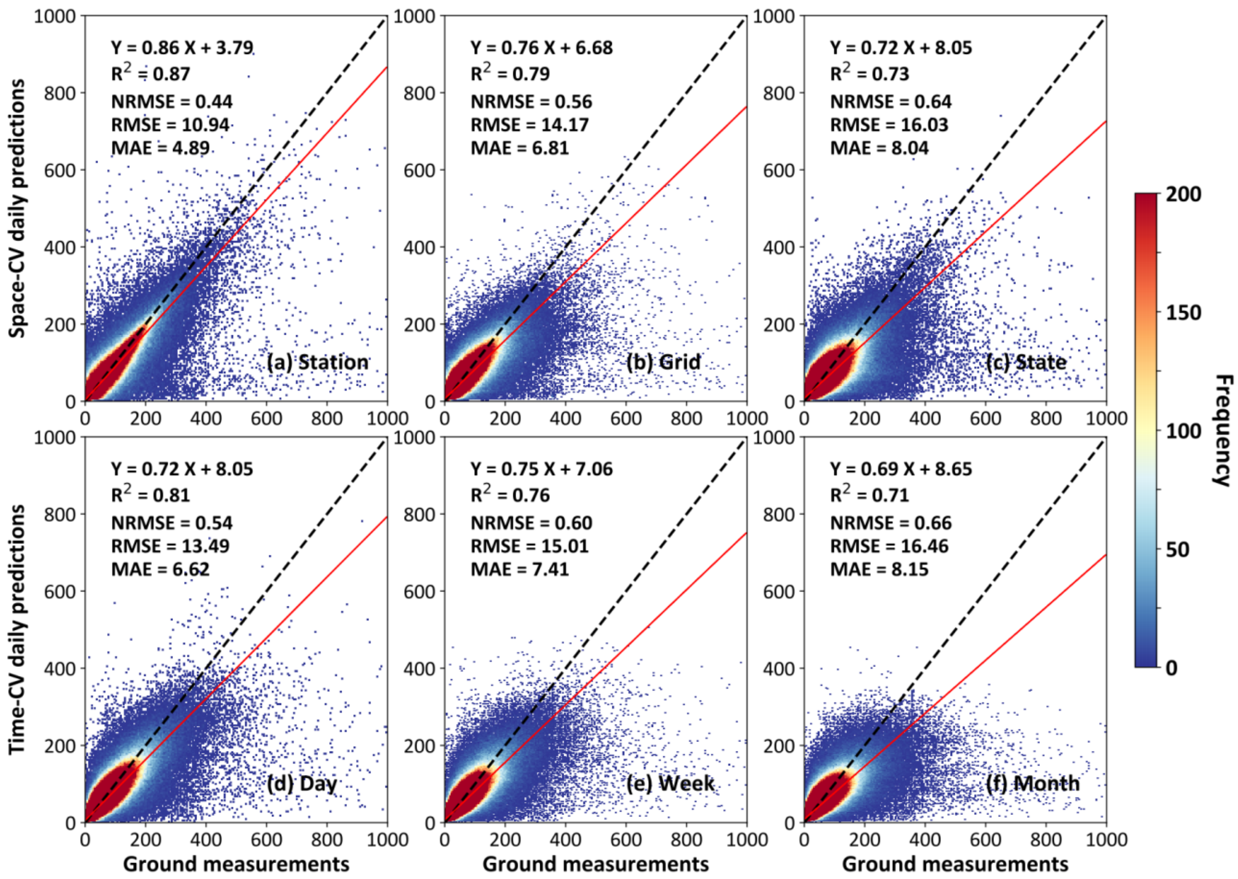

Supplementary Figure 1. Independent spatiotemporal cross-validation results. Density scatterplots of spatial (a) out-of-station, (b) out-of-grid, (c) out-of-state, and temporal (d) out-of-day, (e) out-of-week, and (f) out-of-month cross-validation results of daily $PM_{2.5}$ predictions (unit: μg m^{-3}) against ground-based measurements (unit: μg m^{-3}) at all monitoring stations from 2017 to 2022 over land (number of samples = 7,089,428). Black dashed lines represent 1:1 lines, and red solid lines represent best-fit lines from linear regression. The linear relation, coefficient of determination (R^2), root-mean-square error (RMSE), normalized RMSE (NRMSE), and mean absolute error (MAE) are given in each panel.

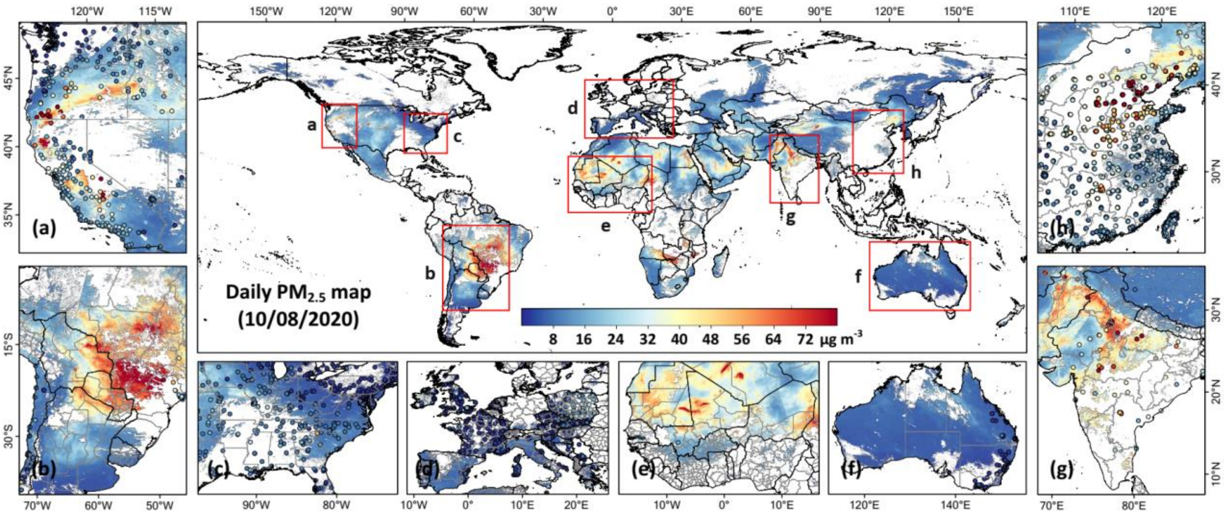

Supplementary Figure 2. Example of a daily $PM_{2.5}$ global map without satellite AOD gap filling. Spatial distribution of global $PM_{2.5}$ concentrations (unit: μg m^{-3}) without AOD gap filling on 8 October 2020. Zoomed-in regions show $PM_{2.5}$ concentrations (unit: μg m^{-3}) measured at monitoring sites (colored dots) over the (a) western United States, (b) central South America, (c) eastern United States, (d) Europe, (e) northwestern Africa, (f) Australia, (g) India, and (h) eastern China. Thin black lines represent country boundaries or shorelines, and gray lines represent state or provincial boundaries. The maps were created using ESRI ArcGIS Pro 3.0.1.

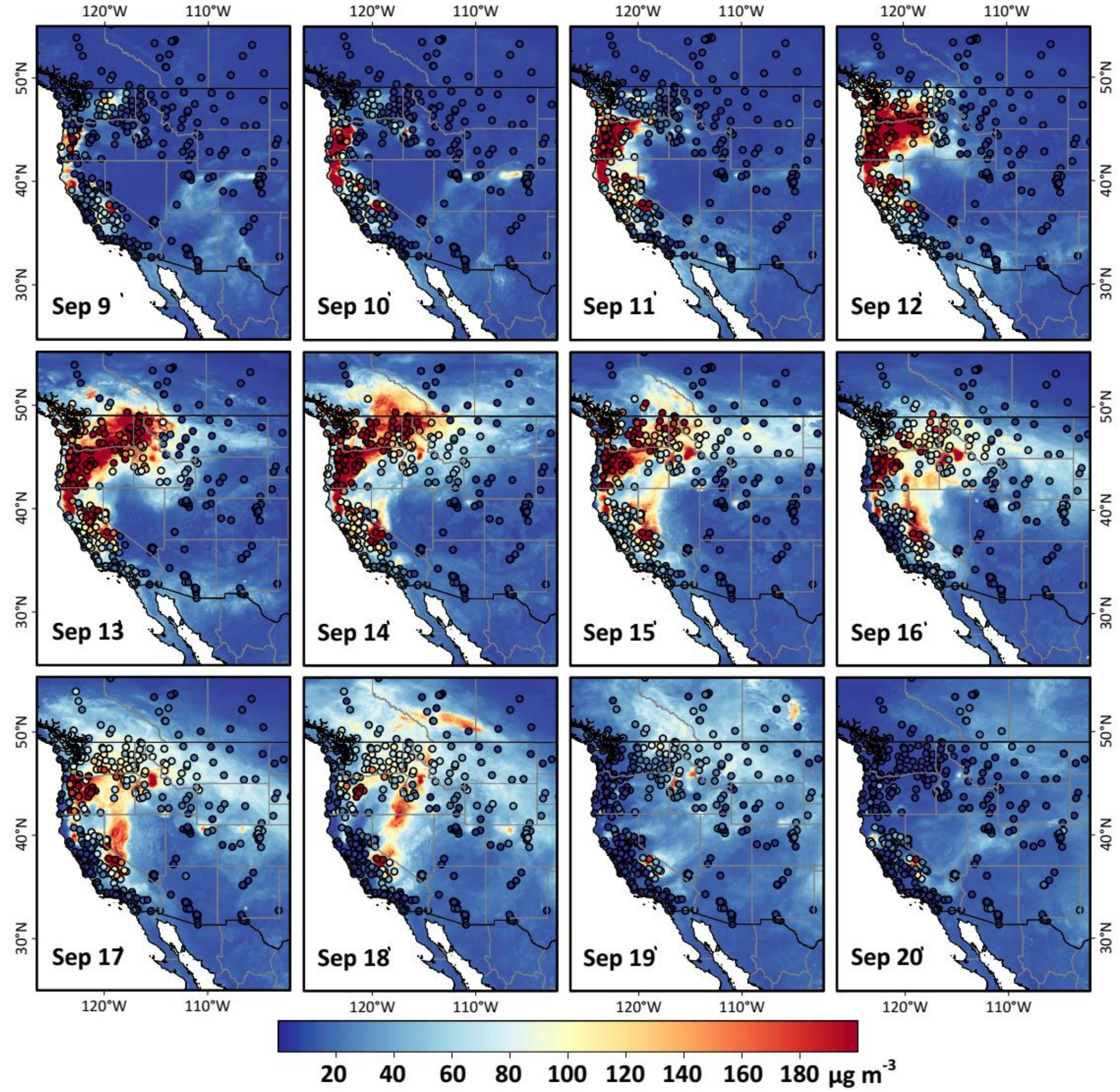

Supplementary Figure 3. $PM_{2.5}$ pollution during a wildfire event in western North America. Spatial distributions of our model-derived (background shading) and ground-measured (dots) daily $PM_{2.5}$ concentrations (unit: $\mu g\ m^{-3}$) during a severe wildfire event that occurred from 9 September to 20 September 2020 in western North America.

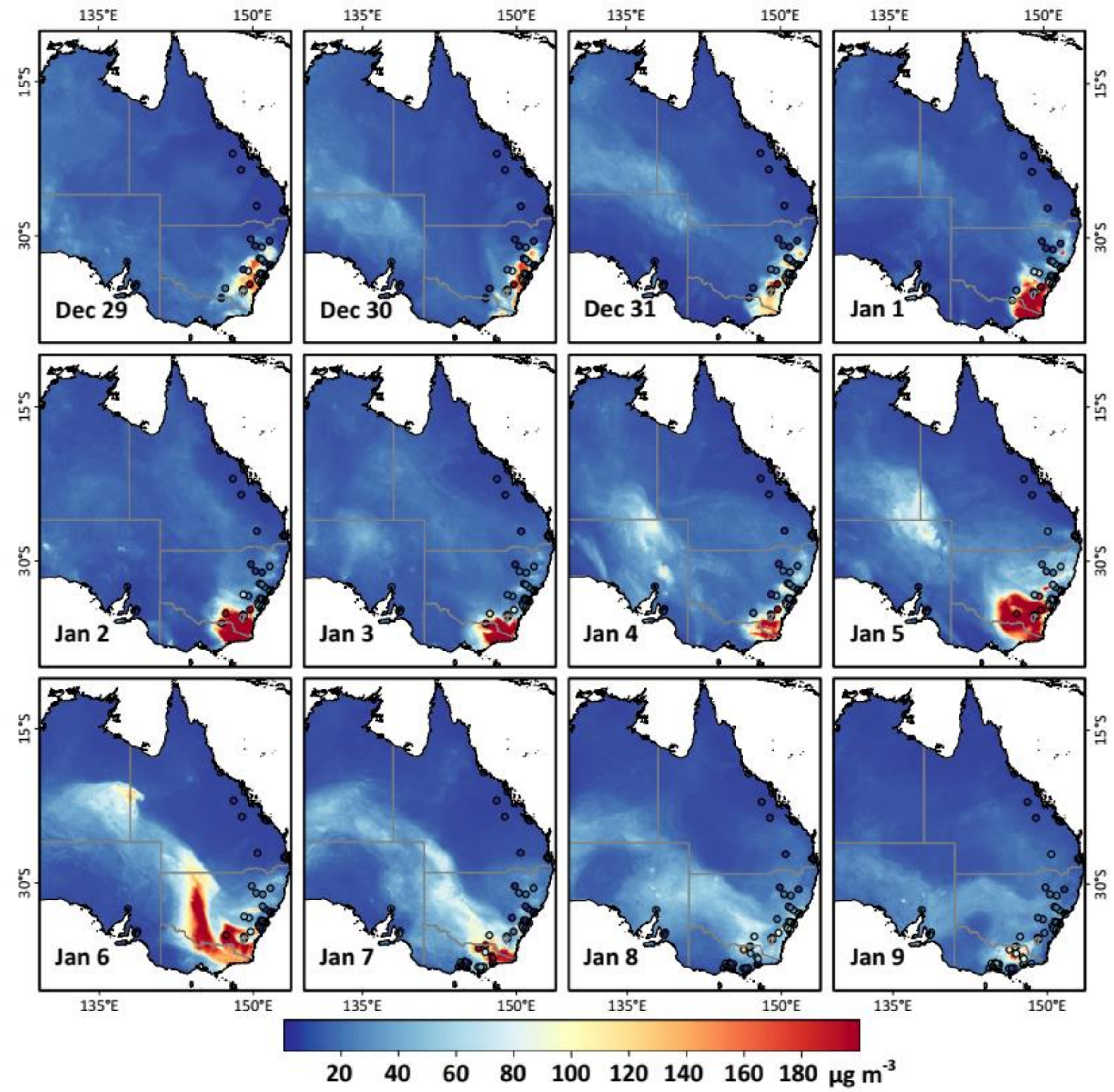

Supplementary Figure 4. $PM_{2.5}$ pollution during a wildfire event in eastern Australia. Spatial distributions of our model-derived (background shading) and ground-measured (dots) daily $PM_{2.5}$ concentrations (unit: $\mu g\ m^{-3}$) during a severe wildfire event that occurred from 29 December 2019 to 9 January 2020 in eastern Australia.

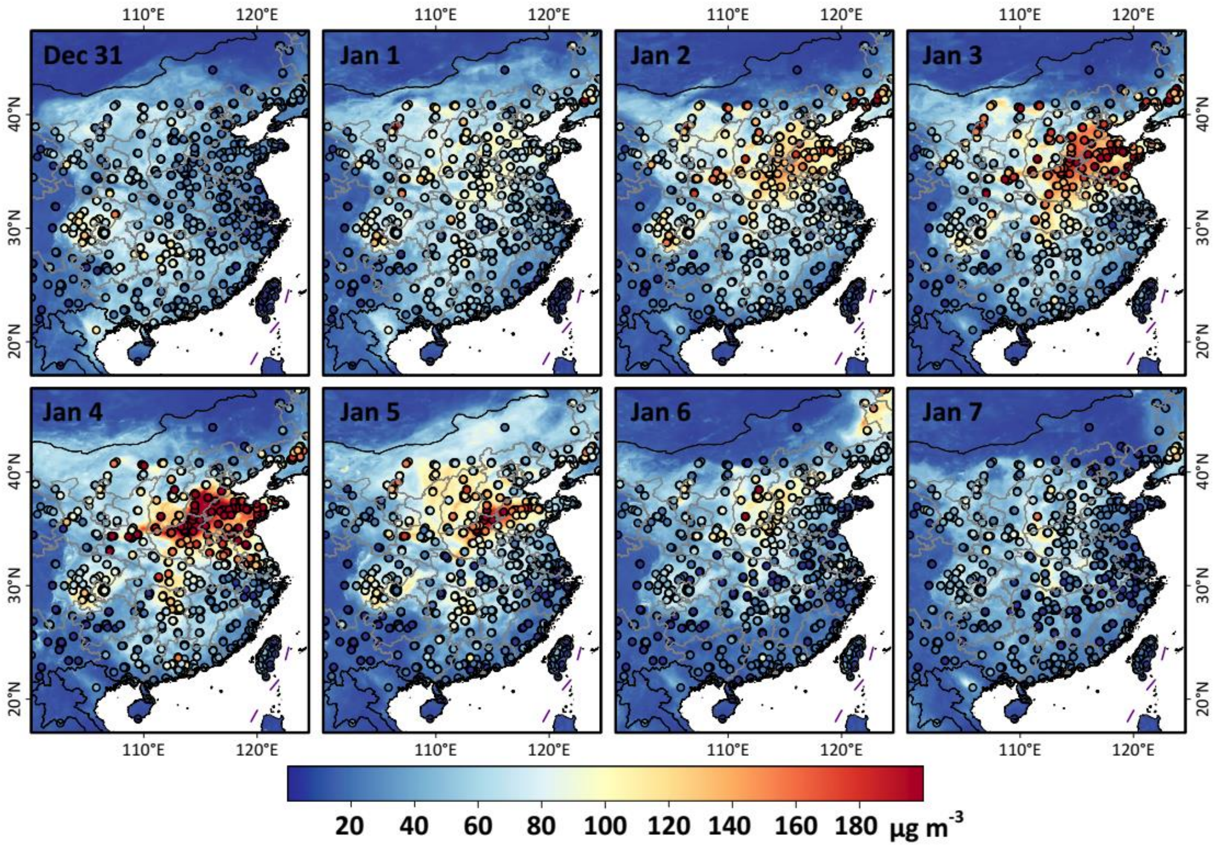

Supplementary Figure 5. $PM_{2.5}$ pollution during a severe haze event in eastern China. Spatial distributions of our model-derived (background shading) and ground-measured (dots) daily $PM_{2.5}$ concentrations (unit: μg m^{-3}) for a typical example of a severe haze event that occurred from 31 December 2019 to 7 January 2020 in eastern China.

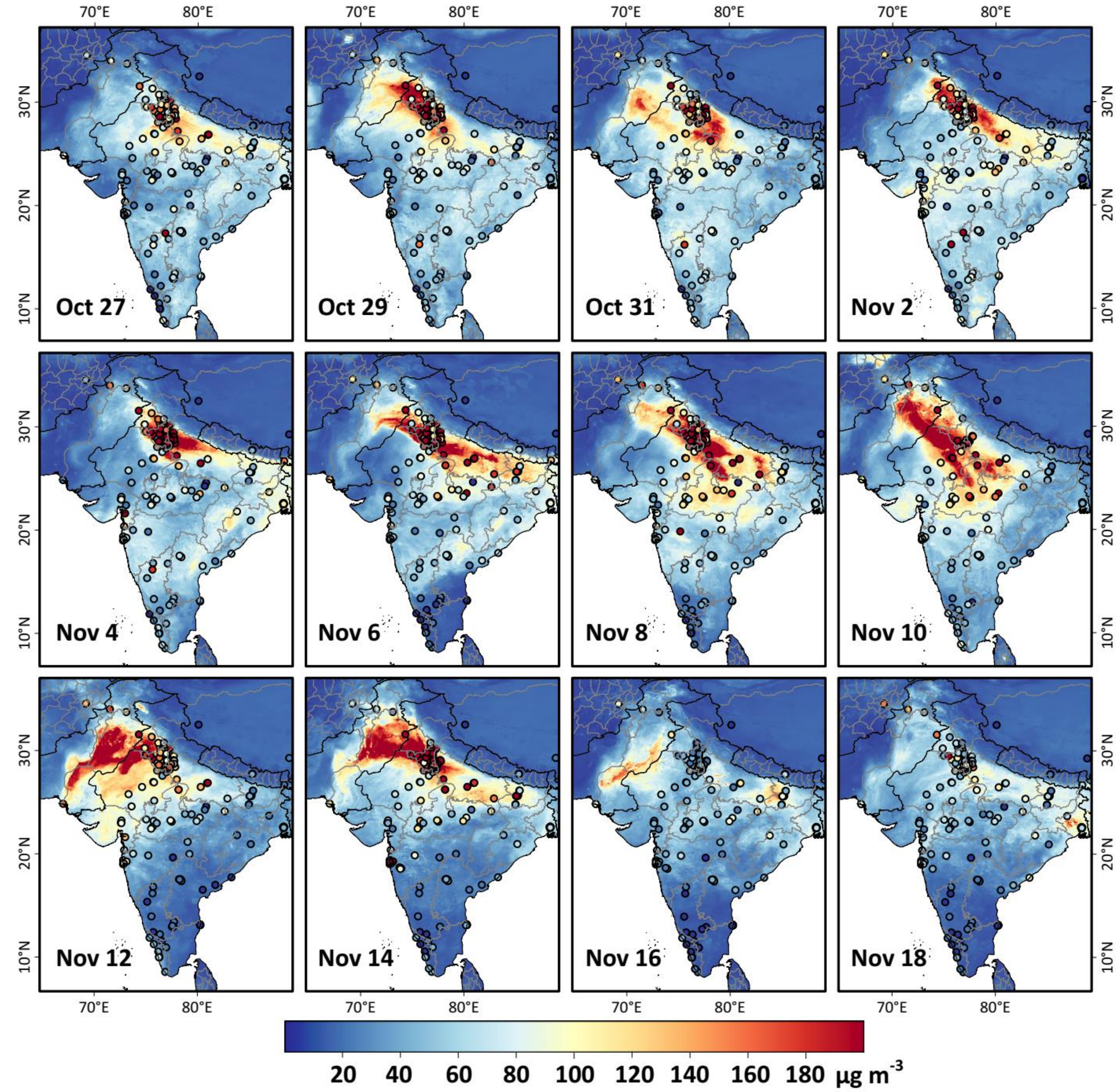

Supplementary Figure 6. $PM_{2.5}$ pollution during a severe haze event in South Asia. Spatial distributions of our model-derived (background shading) and ground-measured (dots) daily $PM_{2.5}$ concentrations (unit: μg m^{-3}) during a severe haze event that occurred from 27 October to 18 November 2020 in South Asia.

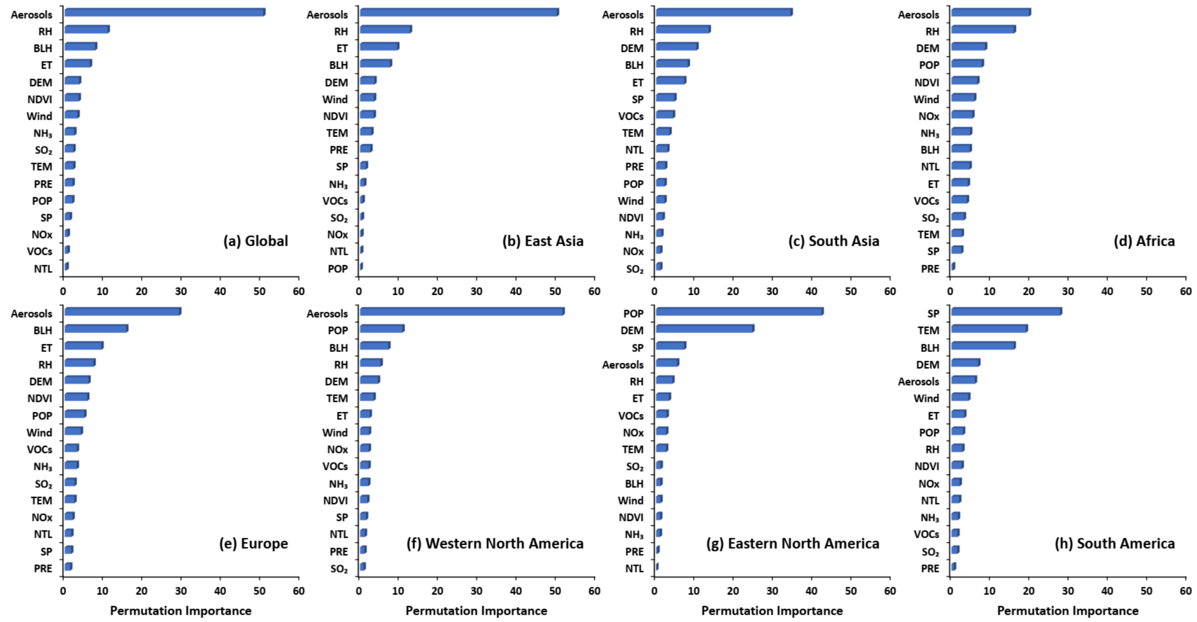

Supplementary Figure 7. Daily $PM_{2.5}$ driving factor analysis with XAI. Driving factor analysis of daily $PM_{2.5}$ pollution (a) on a global scale and within specific localized custom regions: (b) East Asia, (c) South Asia, (d) Africa, (e) Europe, (f) Western North America, (g) Eastern North America, and (h) South America, using Explainable Machine Learning (XAI), with sorted permutation importance scores for each feature. Refer to Supplementary Table 4 for abbreviation definitions.

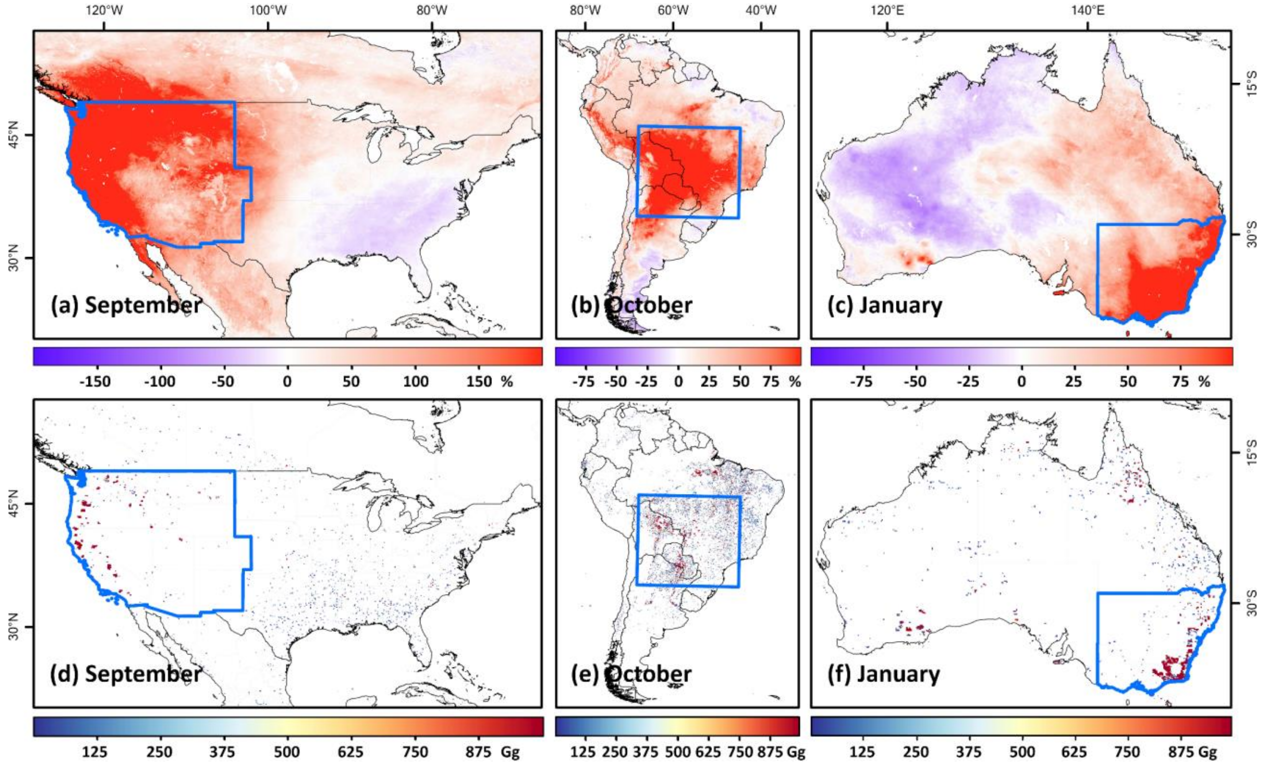

Supplementary Figure 8. $PM_{2.5}$ and fire emission differences between El Niño and non-El-Niño years. Regional relative differences (unit: %) in $PM_{2.5}$ concentrations between the El Niño year (2020) during a month with the most wildfire records and normal years (2018–2019) during the same months for (a) North America in September, (b) South America in October, and (c) Australia in January. Monthly fire emissions (unit: Gg) in 2020 from the Fire Energetics and Emissions Research (FEER) database in these regions and months are shown in (d-f). Blue boundaries represent the defined regions of the western United States, central South America, and southeastern Australia. The maps were created using ESRI ArcGIS Pro 3.0.1.

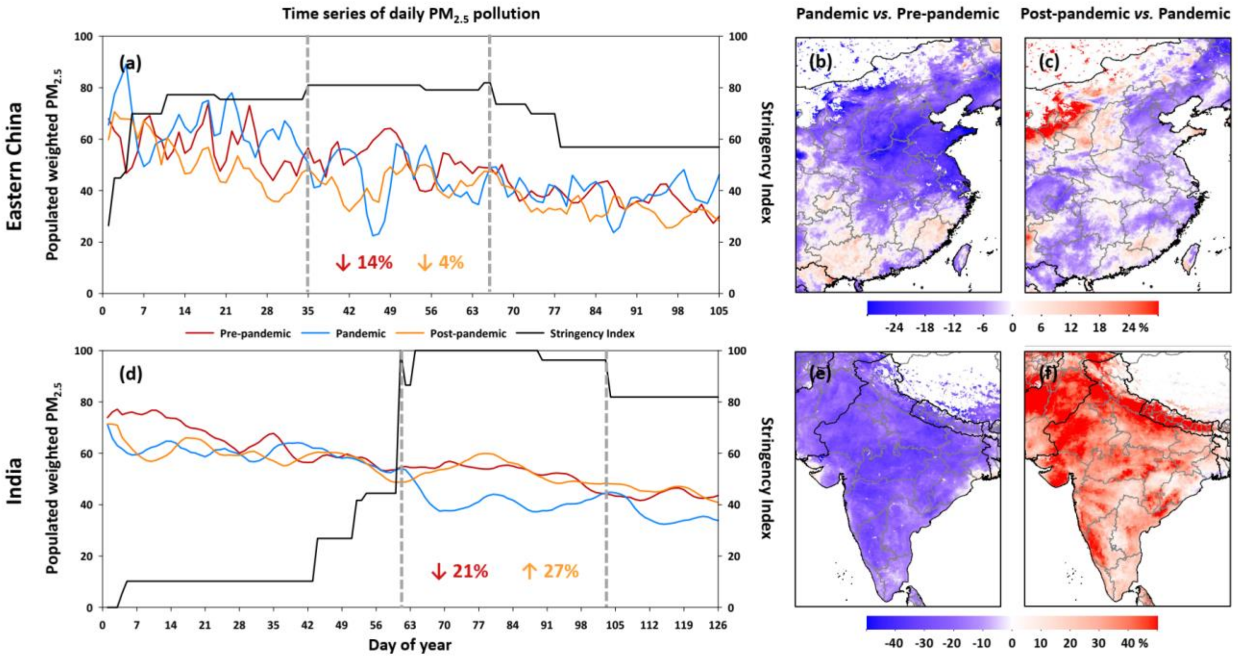

Supplementary Figure 9. Short-term impact of the COVID-19 lockdown on air quality. Time series of 7-day moving average daily population-weighted $PM_{2.5}$ concentrations (unit: µg m^{-3}), and relative differences (unit: %) in $PM_{2.5}$ concentrations during the same strictest lockdown period comparing the pre-pandemic (2018–2019) and post-pandemic (2021–2022) eras to the pandemic year (2020) in (a-c) China and (d-f) India. The time interval between gray dashed lines in (a) and (d) indicates the most stringent lockdown period, determined by the Oxford Coronavirus Government Response Tracker (OxCGRT) stringency index (indicated by the black solid lines in a & d).

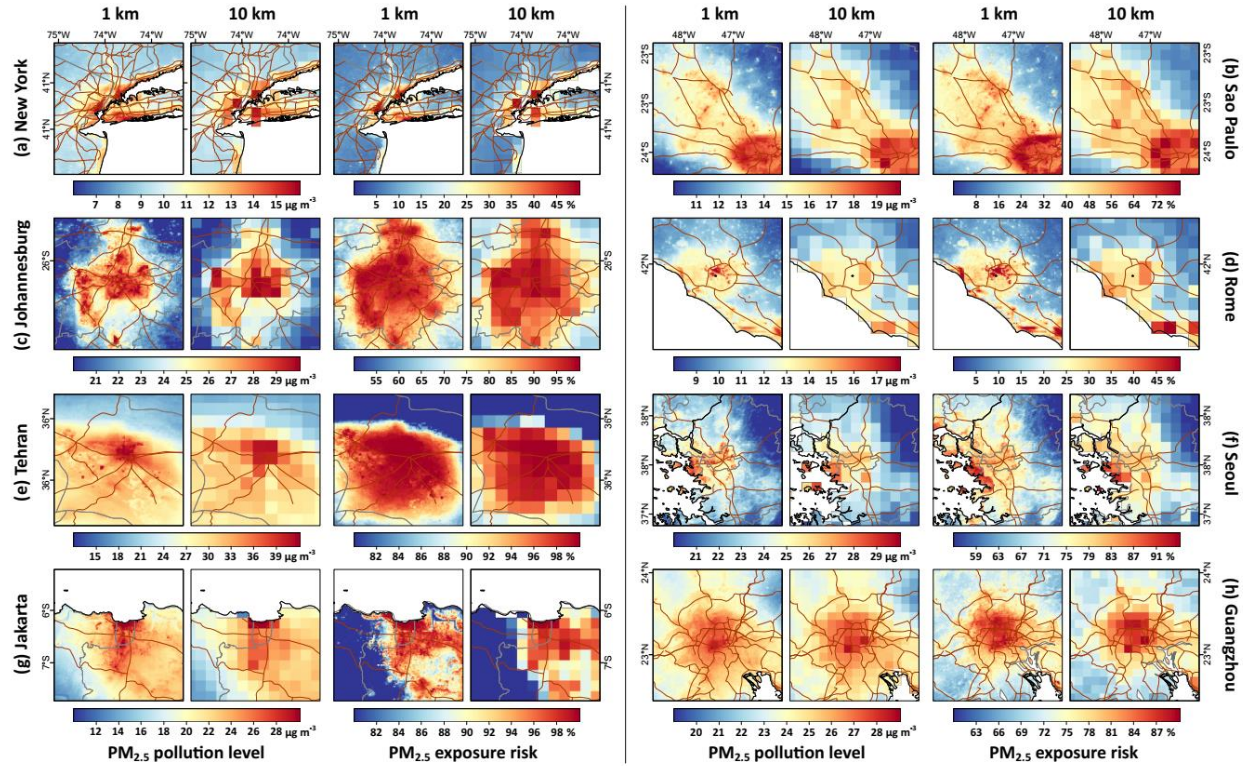

Supplementary Figure 10. Comparing $PM_{2.5}$ pollution levels and exposure risks at 1 and 10 km resolutions. Spatial comparisons of $PM_{2.5}$ pollution level (unit: μg m^{-3}) and exposure risk (unit: %) at 1 km and 10 km spatial resolutions in major cities: (a) New York, United States; (b) Sao Paulo, Brazil; (c) Johannesburg, South Africa; (d) Rome, Italy; (e) Tehran, Iran; (f) Seoul, South Korea; (g) Jakarta, Indonesia, and (h) Guangzhou, China. Solid brown lines represent roads.

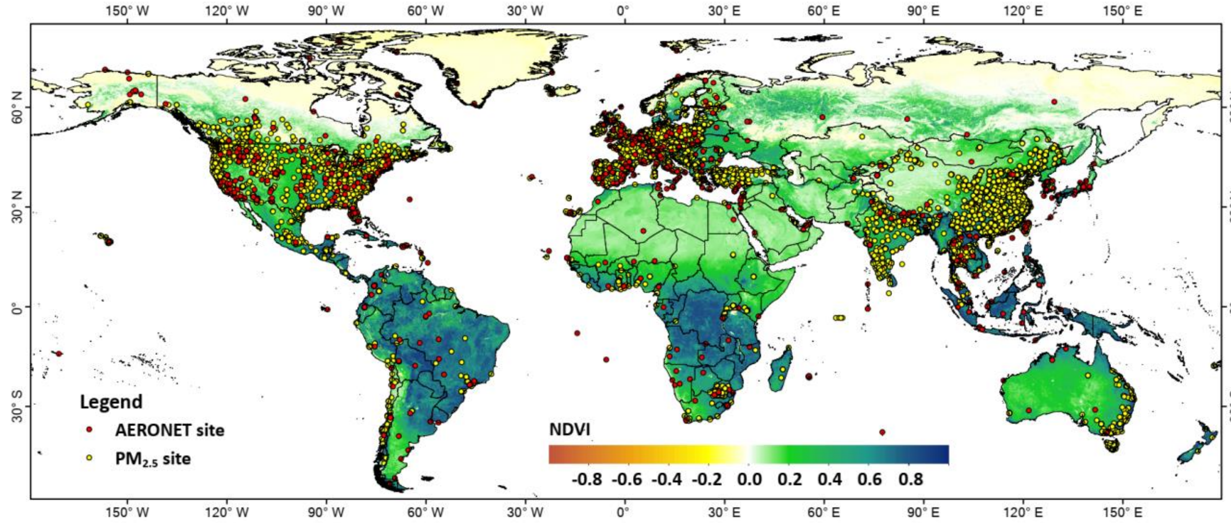

Supplementary Figure 11. Locations of AERONET and $PM_{2.5}$ monitoring stations. Spatial distributions of ground AERONET (red dots) and $PM_{2.5}$ (yellow dots) monitoring stations used in this study. The background map is the NDVI from the MODIS vegetation index product at a 1 km spatial resolution. The map was created using ESRI ArcGIS Pro 3.0.1.

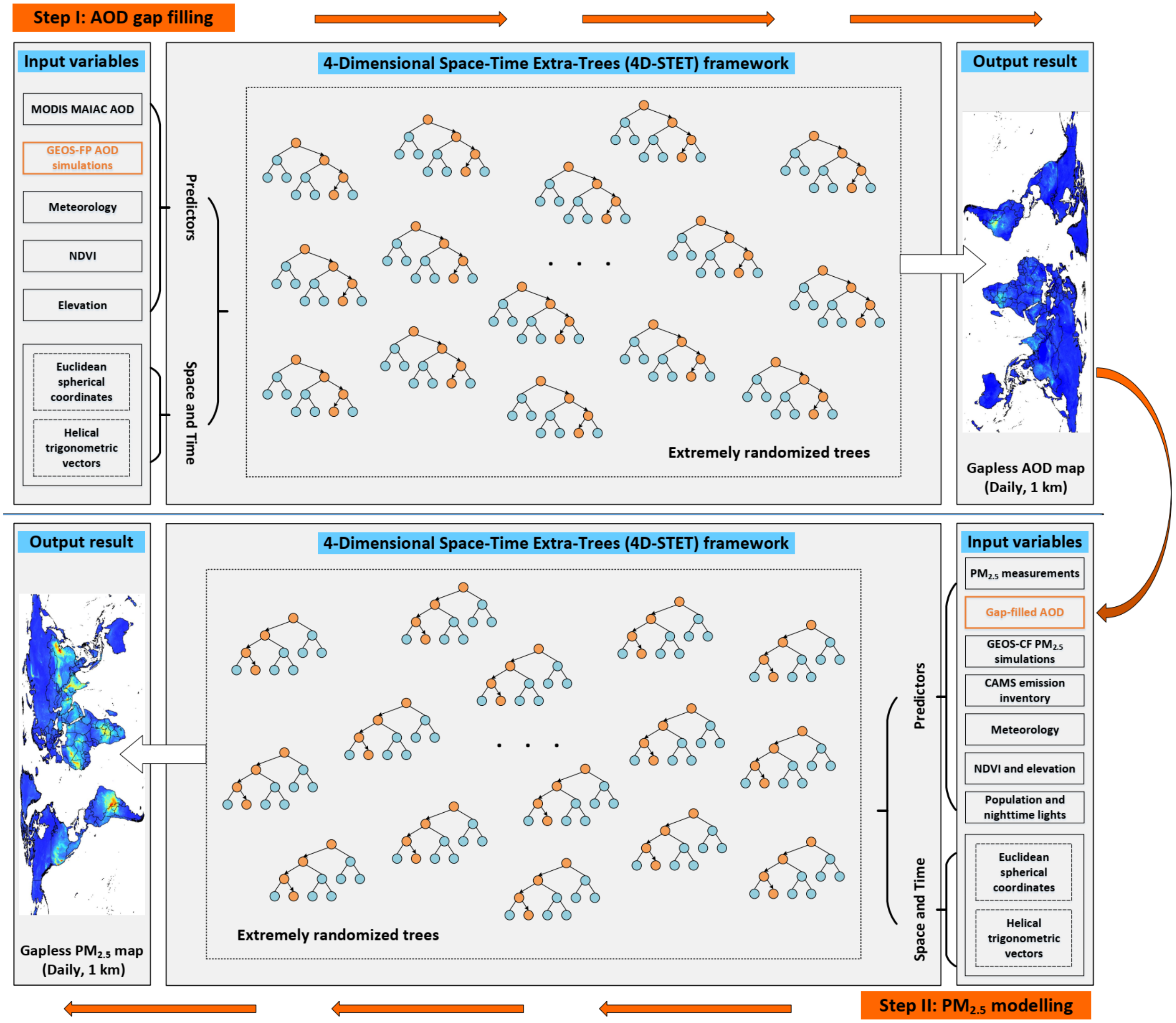

Supplementary Figure 12. 4D-STET framework. Schematic framework of the 4-Dimensional Space-Time Extra-Trees (4D-STET) model developed in this study.

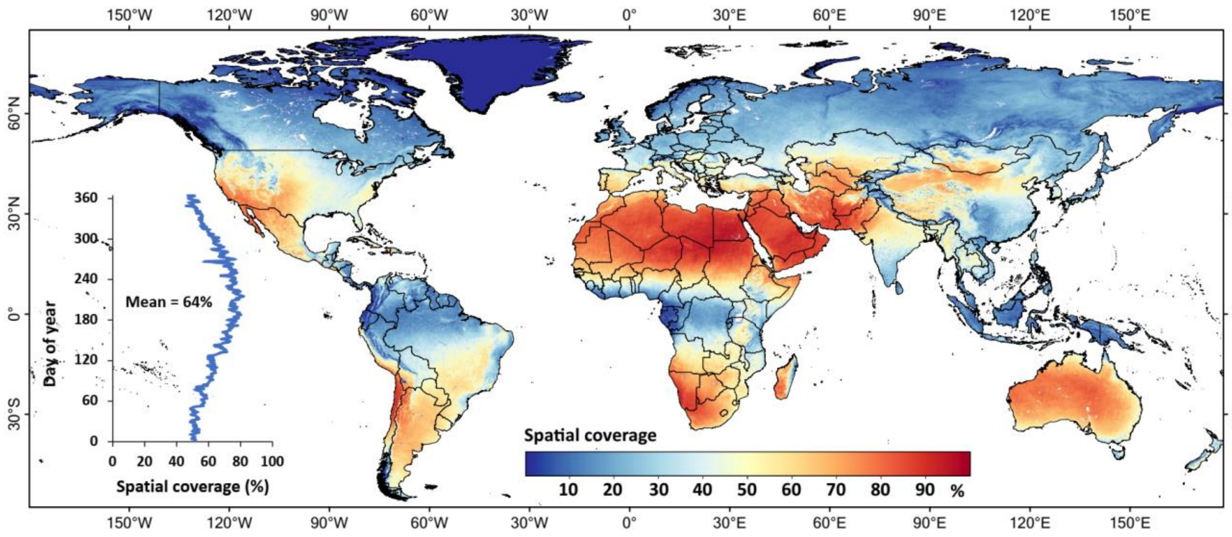

Supplementary Figure 13. Spatial coverage of daily MAIAC AOD retrievals. Spatial distribution of coverage (unit: %) of daily MAIAC AOD retrievals over land, where the insert plot shows the daily times series of spatial coverage. The map was created using ESRI ArcGIS Pro 3.0.1.

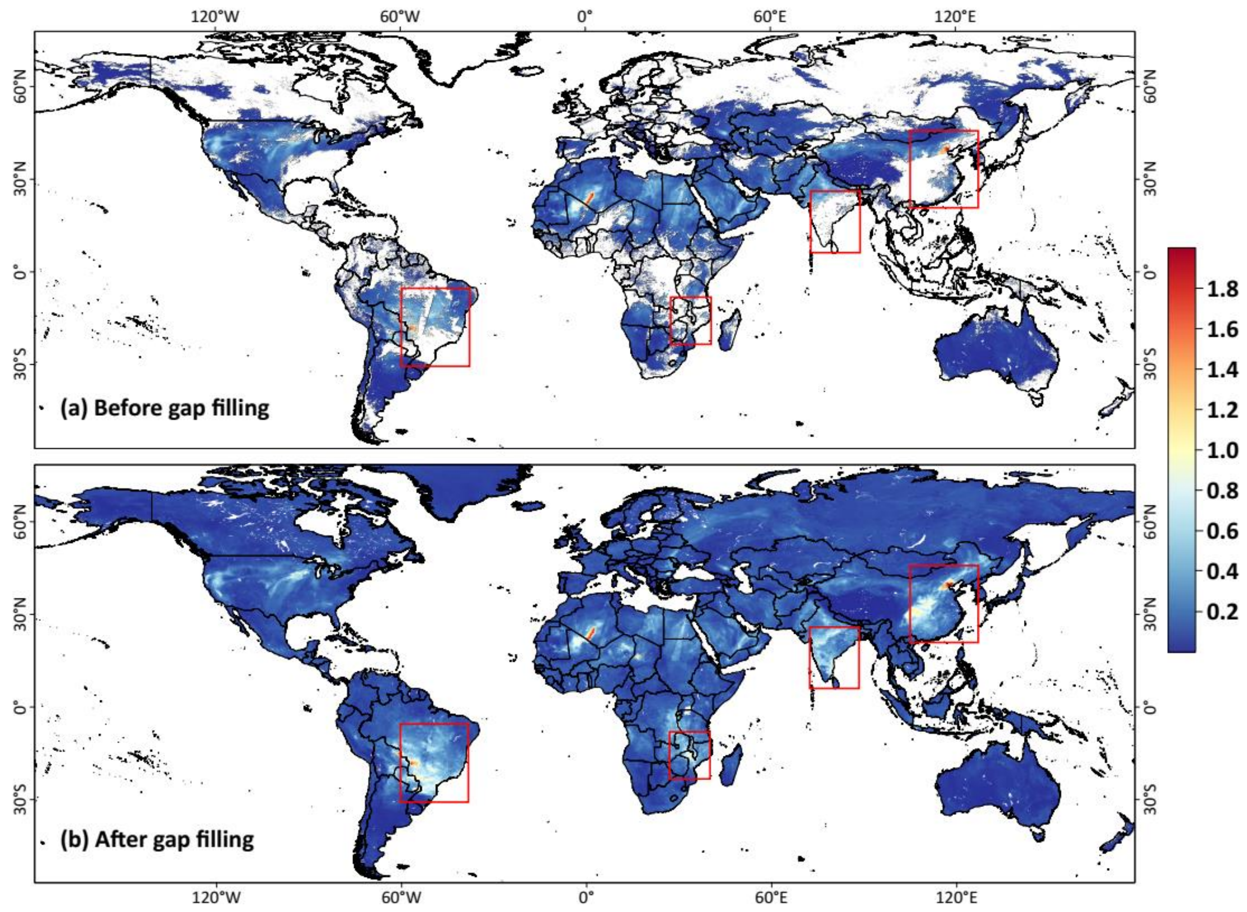

Supplementary Figure 14. Before and after satellite AOD gap filling. Comparison of spatial patterns of daily AOD (a) before and (b) after gap filling over land on an individual day. Areas outlined in red show how gap filling reveals the presence of high AODs previously undetected. The maps were created using ESRI ArcGIS Pro 3.0.1.

Supplementary Table 1. Statistics of continent-stratified cross-validation results of the 4D-STET model in predicting daily $PM_{2.5}$ levels from 2017 to 2022.

Continent	Number of stations	CV-R^2	RMSE (μg m^{-3})	NRMSE	MAE (μg m^{-3})
North America	2729	0.76	4.62	0.52	2.53
South America	276	0.54	14.04	0.66	8.12
Europe	2064	0.75	6.18	0.46	3.21
Africa	469	0.60	15.95	0.67	9.32
Asia	3613	0.89	13.39	0.29	7.70
Oceania	323	0.73	6.31	0.97	2.09

CV-R^2: coefficients of determination for cluster cross-validation stratified by continent

Supplementary Table 2. Top 20 countries with sorted daily exposure risks, showing the percentage of days surpassing the WHO-recommended short-term air quality guideline (S-AQG) level and interim targets (S-IT4 – S-IT1) in 2022.

Rank	Country	Region	S-AQG	S-IT4	S-IT3	S-IT2	S-IT1
1	Kuwait	Middle East	100.0	95.1	72.1	45.8	17.3
2	Pakistan	South Asia	100.0	92.3	67.7	32.6	0.5
3	India	South Asia	100.0	76.7	64.1	22.7	0.0
4	China	East Asia	100.0	62.7	28.2	9.0	0.3
5	Qatar	Middle East	99.7	93.2	67.7	42.2	9.6
6	Saudi Arabia	Middle East	99.7	86.0	48.5	26.3	2.2
7	Iran	Middle East	99.2	45.2	3.6	0.5	0.0
8	Egypt	Middle East	98.6	41.9	12.6	4.7	0.5
9	Bahrain	Middle East	97.5	69.9	36.4	10.1	0.5
10	Iraq	Middle East	96.2	63.8	30.1	11.5	2.2
11	United Arab Emirates	Middle East	95.6	72.1	32.3	10.4	0.3
12	Sudan	North Africa	94.8	44.4	20.3	9.3	2.2
13	Nepal	South Asia	93.7	64.4	29.0	5.2	0.0
14	Niger	North Africa	93.4	69.3	51.2	38.4	17.0
15	Bangladesh	South Asia	91.5	69.0	43.0	28.2	7.4
16	Oman	Middle East	91.2	47.9	15.1	3.0	0.0
17	Afghanistan	West Asia	90.4	29.6	1.1	0.5	0.0
18	Senegal	North Africa	89.6	55.9	21.4	6.3	0.3
19	Nigeria	North Africa	86.8	61.9	35.6	24.1	7.9
20	Congo	Central Africa	84.4	31.8	0.5	0.0	0.0

Supplementary Table 3. Top 20 major cities (defined as urban agglomerations with populations greater than 300,000 reported by the World Urbanization Prospects), showing the percentage of days surpassing the WHO-recommended short-term air quality guideline (S-AQG) level and interim targets (S-IT4 – S-IT1) in 2022.

Rank	City	Country	S-AQG	S-IT4	S-IT3	S-IT2	S-IT1
1	Riyadh*	Saudi Arabia	100.0	99.1	86.1	65.6	35.1
2	Baghdad*	Iraq	100.0	97.8	82.8	60.5	27.7
3	Lahore	Pakistan	100.0	96.7	84.0	69.3	43.9
4	Peshawar	Pakistan	100.0	94.6	71.6	40.5	11.0
5	Faisalabad	Pakistan	100.0	94.4	82.0	61.2	22.9
6	Sargodha	Pakistan	100.0	94.2	75.9	47.3	6.8
7	Rawalpindi	Pakistan	100.0	94.0	66.9	37.9	11.9
8	Gujranwala	Pakistan	100.0	94.0	78.3	57.7	20.2
9	Mardan	Pakistan	100.0	93.9	67.7	38.3	9.6
10	Sialkot	Pakistan	100.0	93.9	71.6	42.6	6.8
11	Gujrat	India	100.0	93.8	72.6	43.8	9.1
12	Tehran*	Iran	100.0	92.8	44.9	21.7	2.2
13	Wah	Pakistan	100.0	92.7	60.3	25.1	3.0
14	Islamabad*	Pakistan	100.0	92.0	61.5	31.8	9.5
15	Kano	Nigeria	100.0	91.8	72.1	60.4	31.7
16	Indore	India	100.0	81.5	57.1	23.8	1.2
17	Karachi	Pakistan	99.7	89.4	56.6	26.9	4.1
18	Khartoum*	Sudan	99.7	77.5	38.7	27.7	10.6
19	Lanzhou	China	99.7	70.6	39.4	17.8	1.3
20	Lima*	Peru	99.7	65.2	9.0	0.3	0.0

Note that * represents the capital of the country.

Supplementary Table 4. Summary of datasets and sources used in this study.

Dataset	Abbreviation	Content	Unit	Spatial Resolution	Temporal Resolution	Data Source
$PM_{2.5}$	$PM_{2.5}$	$PM_{2.5}$ measurements	μg/m^3	in situ	Hourly	OpenAQ, etc.
AOD	SAOD	Satellite AOD (550 nm)	-	1 km × 1 km	Daily	MCD19A2
AOD	MAOD	Model AOD (550 nm)	-	0.25 ° × 0.3125 °	3-hour	GEOS-FP
$PM_{2.5}$	$MPM_{2.5}$	Model $PM_{2.5}$	μg/m^3	0.25 ° × 0.25 °	Hourly	GEOS-CF
Precursor	NH_3	Ammonia	Mg/grid	0.1 ° × 0.1 °	Monthly	CAMS
	NO_x	Nitrogen oxides	Mg/grid			
	SO_2	Sulfur dioxide	Mg/grid			
	VOCs	Volatile organic compounds	Mg/grid			
Meteorology	TEM	Temperature	K	0.1 ° × 0.1 °	Hourly	ERA5
	WU	u-component of wind	m/s			
	WV	v-component of wind	m/s			
	SP	Surface pressure	hPa			
	PRE	Precipitation	mm			
	ET	Evaporation	mm			
	BLH	Boundary-layer height	m	0.25 ° × 0.25 °		
	RH	Relative humidity	%			
Population	POP	Population	-	1 km × 1 km	Annual	WorldPop
Economy	NTL	Nighttime lights		500 m × 500 m	Monthly	VIIRS
Land cover	NDVI	NDVI	-	1 km × 1 km	Monthly	MOD13A3
Terrain	DEM	Surface elevation	m	90 m × 90 m	-	SRTM

Supplementary Table 5. Comparison of model performance using multidimensional and traditional spatiotemporal information from 2017 to 2022 as input variables.

CV method	New approach		Traditional approach	
	$CV\text{-}R^2$	RMSE ($\mu g\ m^{-3}$)	$CV\text{-}R^2$	RMSE ($\mu g\ m^{-3}$)
Sample-based CV	0.91	9.20	0.88	10.56
Station-based CV	0.87	10.94	0.84	12.26
Grid-based CV	0.79	14.17	0.75	15.48
State-based CV	0.73	16.03	0.69	17.17
Day-based CV	0.81	13.49	0.78	14.54
Week-based CV	0.76	15.01	0.74	15.75
Month-based CV	0.71	16.46	0.69	17.29

Supplementary Note 1: Unravelling daily $PM_{2.5}$ driving factors with XAI

$PM_{2.5}$ concentrations are influenced by a diverse range of factors, both natural processes and human activities, which can vary significantly across different regions of the globe. To gain deeper insights into the driving factors impacting daily $PM_{2.5}$ pollution, we employed Explainable Machine Learning (XAI) techniques. Adopted here is the Permutation Importance (PI) method, which involves randomly permuting the values of individual features in the dataset and quantifying the resulting drop in model performance [1]. A larger drop indicates a higher level of importance for that specific feature. The PI method is a model-independent method and has no preference for continuous variables or high-cardinality categorical variables, reflecting the generalization ability of these variables. Additionally, it is particularly valuable when dealing with data shifts, enabling a more accurate measurement of each feature's contribution [2].

Our findings indicate that globally, aerosols (including satellite AOD and modelled $PM_{2.5}$) have the most significant contribution, accounting for more than half of the score (~51%) (Figure S7). Additionally, they dominate (PI = 20–51%) over vast regions experiencing air pollution [3], including East Asia and South Asia with substantial anthropogenic emissions, Africa with a mixture of dust and biomass burning sources, Europe and the western US with increasing wildfires in recent years [4]. Besides, meteorological conditions play a crucial role in explaining daily $PM_{2.5}$ levels (PI = 20–73%), particularly relative humidity, evaporation, and boundary layer height, which are closely intertwined with the production, hygroscopic growth, and removal of $PM_{2.5}$, as well as the vertical distribution and variations of reactive aerosol particles [5]. Terrain can influence the transport and deposition of air pollution, highlighting their substantial role. By contrast, within clean areas like the eastern US and South America, the driving factors are more intricate, leading to the diminishing importance of aerosols, while the significance of population density, elevation, and meteorological conditions (e.g., air pressure and temperature) increases noticeably. These findings offer an initial interpretable examination of the complex interplay between various factors and daily $PM_{2.5}$ pollution at global and regional scales. Further investigations are warranted to explore the underlying physical mechanisms and chemical reactions for decision making and devising sustainable measures to tackle air pollution challenges.

Supplementary Note 2: 4-Dimensional Space-Time Extra-Trees model

$PM_{2.5}$ varies spatially and has strong seasonal cycles. Air pollution varies considerably over time and space across various scales. Importantly, these variations are often nonlinear. Relying solely on coordinates (e.g., longitude, latitude) and time-based information (e.g., month, day of the year) may not fully capture the spatiotemporal autocorrelations and fluctuations of individual points in both space and time, especially when considering different global hemispheres and the influence of seasonal cycles. Therefore, a new 4-Dimensional Space-Time Extra-Trees (4D-STET) model was developed by optimizing our approach to better capture spatiotemporal information based on polar coordinates [6,7], i.e., the spatial term (Ps) is represented by the unequal autocorrelation and differences of points in space using three spherical coordinates in Euclidean space, i.e., Ps ~ [S_1, S_2, S_3], calculated using latitude (Lat) and longitude (Lon) information of one given point in space as follows:

$$S_1 = \sin\left(2\pi\frac{Lon}{360}\right), \quad (1)$$

$$S_2 = \cos\left(2\pi\frac{Lon}{360}\right)\sin\left(2\pi\frac{Lat}{180}\right), \quad (2)$$

$$S_3 = \cos\left(2\pi\frac{Lon}{360}\right)\cos\left(2\pi\frac{Lat}{180}\right). \quad (3)$$

Additionally, we express the temporal term (Pt) of points using three helix-shaped trigonometric vectors, i.e., Pt ~ [T_1, T_2, T_3], allowing us to incorporate both the daily variations and seasonal cycles of air pollution, calculated using the day of the year (DOY) of one given point in a time series as follows:

$$T_1 = \frac{DOY}{N}, \quad (4)$$

$$T_2 = \cos\left(2\pi\frac{DOY}{N}\right), \quad (5)$$

$$T_3 = \sin\left(2\pi\frac{DOY}{N}\right), \quad (6)$$

where N represents the total number of days in a year.

Supplementary Note 3: Air pollution modelling

Satellite AOD gap filling (Equation 7) and surface $PM_{2.5}$ estimations (Equation 8) were performed and constructed by training the developed 4D-STET model with separate input target and independent variables, expressed as follows:

$$SAOD_{gd} \sim f_{\text{4D-STET}}(MAOD_{gd}, BLH_{gd}, TEM_{gd}, RH_{gd}, WU_{gd}, WV_{gd}, SP_{gd}, NDVI_{gm}, DEM_{g}, Ps_{g}, Pt_{g}) \;, \quad (7)$$

$$PM_{2.5(gd)} \sim f_{\text{4D-STET}}(FAOD_{gd}, MPM_{2.5(gd)}, NH_{4(gm)}, NO_{x(gm)}, SO_{2(gm)}, VOCs_{gm}, BLH_{gd}, TEM_{gd}, RH_{gd}, WU_{gd}, WV_{gd}, SP_{gd}, PRE_{gd}, ET_{gd}, NDVI_{gm}, NTL_{gm}, POP_{gy}, DEM_{g}, Ps_{g}, Pt_{g}) \;, \quad (8)$$

where $SAOD_{gd}$, $MAOD_{gd}$, $FAOD_{gd}$, $PM_{2.5(gd)}$, and $MPM_{2.5(gd)}$ represent combined MAIAC AOD, modeled AOD, gap-filled AOD, measured $PM_{2.5}$, and modeled $PM_{2.5}$, respectively, in grid cell g on day d of the year; BLH_{gd}, TEM_{gd}, RH_{gd}, WU_{gd}, WV_{ugd}, SP_{gd}, PRE_{gt}, and ET_{gt} represent the mean boundary-layer height, temperature, relative humidity, horizontal and vertical components of wind, surface pressure, precipitation, and evaporation, respectively, in grid cell g on day d of the year; $NDVI_{gm}$, NTL_{gm}, $NH_{4(gm)}$, $NO_{x(gm)}$, $SO_{2(gm)}$, and $VOCs_{gm}$ represent the mean normalized vegetation index, nighttime lights, and anthropogenic emissions of ammonia, nitrogen oxides, sulfur dioxide, and volatile organic compounds, respectively, in grid cell g of month d of the year; DEM_{g} represents the surface elevation in grid cell g; POP_{gy} represents the population number in grid cell g of year y; and Ps and Pt represent spatial and temporal information about air pollutants, respectively, in grid cell g.

Supplementary Note 4: Data availability

OpenAQ data is available at https://openaq.org/; China CNEMC $PM_{2.5}$ measurements are available at http://www.cnemc.cn; US EPA $PM_{2.5}$ measurements are available at (https://www.epa.gov); Canada NAPS $PM_{2.5}$ measurements are available at https://data-donnees.ec.gc.ca/data/air/monitor/national-air-pollution-surveillance-naps-program/; European AQ e-Reporting $PM_{2.5}$ measurements are available at https://www.eea.europa.eu/; $PM_{2.5}$ measurements for South Africa, New Zealand, and Brazil are available at https://saaqis.environment.gov.za/, https://www.stats.govt.nz/indicators/pm2-5-concentrations/, and https://energiaeambiente.org.br/qualidadedoar/; MODIS MAIAC AOD and NDVI products are available at https://search.earthdata.nasa.gov/; GEOS-FP and GEOS-FP data are available at https://portal.nccs.nasa.gov/datashare/gmao/; CAMS emission inventory is available at https://ads.atmosphere.copernicus.eu/; ERA5 reanalysis is available at https://cds.climate.copernicus.eu/; WroldPOP global population data is available at https://hub.worldpop.org/; VIIRS nighttime lights is available at https://eogdata.mines.edu/products/vnl/; and SRTM DEM is available at https://www2.jpl.nasa.gov/srtm/.

Supplementary References

1. Fisher, A., Rudin, C. & Dominici, F. All models are wrong, but many are useful: Learning a variable's importance by studying an entire class of prediction models simultaneously. *J. Mach. Learn. Res.* 20 (2019).
2. Tian, Z., Wei, J. & Li, Z. How important is satellite-retrieved aerosol optical depth in deriving surface $PM_{2.5}$ using Machine Learning? *Remote Sens.* **15**, 3780 (2023).
3. Shaddick, G., Thomas, M. L., Mudu, P., Ruggeri, G. & Gumy, S. Half the world's population are exposed to increasing air pollution. *npj Clim. Atmos. Sci.* **3**, 23 (2020).
4. McClure, C. D. & Jaffe, D. A. US particulate matter air quality improves except in wildfire-prone areas. *Proc. Natl. Acad. Sci. USA* **115**, 7901-7906 (2018).
5. Li, Z. *et al.* Aerosol and boundary-layer interactions and impact on air quality. *Natl. Sci. Rev.* **4**, 810-833 (2017).
6. Wei, J. *et al.* Separating daily 1 km $PM_{2.5}$ inorganic chemical composition in China since 2000 via deep learning integrating ground, satellite, and model data. *Environ. Sci. Technol.* doi:10.1021/acs.est.3c00272 (2023).
7. Sun, H. *et al.* Spatial resolved surface ozone with urban and rural differentiation during 1990-2019: A space-time bayesian neural network downscaler. *Environ. Sci. Technol.* **56**, 7337-7349 (2022).
